# Association between 25-hydroxy vitamin D, interleukin-4, and interferon-γ levels and asthma in children with Mycoplasma pneumonia infection

**DOI:** 10.1038/s41598-024-80322-4

**Published:** 2024-11-21

**Authors:** Tao Shen, Tingting Liu, Luke Kong, Yanfang Li

**Affiliations:** 1 Department of Clinical Laboratory, Jincheng People’s Hospital, 1666 Baishui East Street, Jincheng, 048000 Shanxi China; 2 Jincheng Hospital Affiliated to Changzhi Medical College, Jincheng, China

**Keywords:** Mycoplasma pneumoniae, Mycoplasma pneumoniae infection-related asthma, 25-(OH)-D, IL-4, IFN-γ, Microbiology, Diseases

## Abstract

To explore the association between 25-hydroxy vitamin D [25-(OH)-D], interleukin-4 (IL-4), and interferon-γ (IFN-γ) in children with Mycoplasma pneumoniae (MP) infection-related asthma. Logistic analysis was conducted to compare general data in MP asthma and MP non-asthma groups. The level of 25-(OH)-D, IL-4, and IFN-γ were detected and compared between groups. Moreover, the receiver operating characteristic curve (ROC) was applied to test the predictive value of each variable. The results of logistic regression analysis demonstrated that recurrent upper respiratory tract infections and collective living are related to the incidence of MP infection whether with asthma or without asthma. IL-4 and IFN-γ in MP asthma group were significantly higher than those in MP non-asthma group and control group (*p* < 0. 05), whilst 25-(OH)-D and IFN-γ/IL-4 in MP asthma group were significantly lower than those in MP non-asthma group and control group (*p* < 0. 05). ROC curves indicated that the area under the curve (AUC) of 25-(OH)-D, IL-4, IFN-γ, IFN-γ/IL-4, and joint detection are 0.765, 0.780, 0.853, 0.638, and 0.912 in diagnosis of MP infection-related asthma, and sensitivity and specificity of joint detection are both greater than 95%. For children with MP infection-related asthma, the level of IL-4 and IFN-γ is upregulated, while 25-(OH)-D is downregulated. The joint detection of 25-(OH)-D, IL-4, IFN-γ, and IFN-γ/IL-4 may improve diagnostic capabilities of MP infection-related asthma.

## Introduction

Mycoplasma pneumoniae (MP) is a common pathogen leading to respiratory infection in children, mainly manifesting as fever, dry cough, and asthma^1^. Asthma, as an important clinical manifestation of MP infection, is a heterogeneous disease characterized by chronic airway inflammation and airway hyperresponsiveness, the main clinical manifestations comprise recurrent wheezing, coughing, shortness of breath, and chest tightness, which often occurs or worsens at night and/or in the morning^2^. Immune system disorder and airway inflammation are regarded as the pathological and physiological basis of MP infection-related asthma^3–5^. Numerous studies have demonstrated that various factors such as 25-(OH)-D^6–8^, cytokines^9–11^, signal transducer activity^12–14^, and eosinophilic infiltration^15–17^ can effectively promote or alleviate airway inflammation.

It is acknowledged that 25-(OH)-D, an essential fat-soluble vitamin for the growth and development of body, participates in calcium and phosphorus metabolism^18,19^. The anti-infectious immunity function of 25-(OH)-D is also crucial and has been demonstrated by several studies. Specifically, low 25-(OH)-D level is closely associated with increased risk of other pathogens’ infection such as coronavirus^20^, influenza virus^21^, human immunodeficiency virus and hepatitis C virus co-infected^22^, and human papillomavirus^23^. Moreover, the study of Kun, Silei^7^ has suggested that low level of 25-(OH)-D can weaken the immune function of the body and exacerbate the severity of MP infection, nevertheless, whether 25-(OH)-D has a significant effect on MP infection-related asthma still needs further exploration.

The immune system of body is immediately triggered after MP invasion, leading to many kinds of intrapulmonary injury and extrapulmonary injury including cardiovascular system, central nervous system, respiratory system, and urinary system^24^. During the entire course of infection, the role of helper T (Th) cells comprising Th1 and Th2 cells is remarkable, achieving a dynamic equilibrium between Th1 and Th2 cells is crucial to maintain normal immune function in the body^25^. Specifically, Th1 cells can participate in cellular immune responses to resist infection via secreting IFN-γ that may promote the release of pro-inflammatory factors, enhance the phagocytic function of monocytes, and inhibit lymphocyte apoptosis^26–28^. Regarding the main function of Th2 cells, it is to regulate humoral immunity via secreting IL-4, it is worth noting that, however, excessive secretion of IL-4 can contribute to the production of a large amount of specific IgE and the aggregation of eosinophil aggregation in the airway, leading to airway hyperresponsiveness and even irreversible tissue damage^29–31^. However, the significance of IFN-γ and IL-4 in children with MP infection-related asthma is not fully elucidated.

In this study, we try to analyze the association between 25-(OH)-D, IL-4, IFN-γ, and IFN-γ/IL-4 in children with MP infection-related asthma, providing the theoretical basis for the prevention, early diagnosis, and treatment of MP infection.

## Marerials and methods

### Research subjects

In the pediatric department of Jincheng People’s Hospital from December 2022 to December 2023, a total of 78 children with MP infection were selected as the case group and were further divided into MP asthma group (*n* = 37) and MP non-asthma group (*n* = 41) according to the diagnostic criteria for asthma in the “Guidelines for Diagnosis and Prevention of Childhood Bronchial Asthma”^32^. Specifically, children with recurrent and persistent coughing or wheezing were divided into the MP asthma group, while those without the aforementioned symptoms were classified as the MP non-asthma group. Meanwhile, 34 children with physical examination in the same period who do not have any physical or mental illnesses were selected as the control group. Specifically, MP asthma group, including 20 males (54.05%), 17 females (45.95%), aged (4.97 ± 0.95) years old. 41 children with MP infection without asthma symptoms during the same period were assigned as the MP non-asthma group, including 19 males (46.34%), 22 females (53.66%), aged (4.78 ± 0.88) years old. Inclusion criteria: (1) age ranges from 3 to 12 years old, (2) manifestation of acute respiratory infection, (3) existence of pulmonary lesions, (4) MP IgM titer is greater than 1:160, or MP IgM is from negative to positive within one week, or MP DNA or RNA test result is positive, (5) the case has undergone formal treatment and clinical data is complete. Exclusion criteria: (1) the case with immunodeficiency diseases, severe liver and kidney dysfunction, and blood system diseases, etc., (2) the case is within 14 days of surgeries or injuries. Meanwhile, 34 children who underwent health examination were selected as the control group, comprising 18 males (52.94%), 16 females (47.06%), aged (4.67 ± 0.73) years old. This study has been approved by the Medical Ethics Committee of Jincheng People’s Hospital.

## Methods

### Data collection

General information of all research subjects comprising gender, age, body mass index (BMI), history of recurrent upper respiratory tract infections, and collective living (have attended kindergarten or elementary school before the onset of the disease) was collected from the electronic medical record system of Jincheng People’s Hospital.

### Standard protocol approvals, registrations, and patient consents

All procedures performed in studies involving human participants were in accordance with the ethical standards of the institutional research committee and with the 1964 Helsinki declaration and its later amendments or comparable ethics standards. The informed consent was obtained from participating children parent.

### Ethical approval

All procedures performed in studies involving human participants were in accordance with the ethical standards of the institutional research committee and with the 1964 Helsinki declaration and its later amendments or comparable ethics standards. Approval of the ethics committee was obtained from Jincheng People’s Hospital.

### Laboratory examination

Collected 5ml venous blood on an empty stomach, and centrifuged at 3000r/min for 10 minutes. Then, collected serum to detect IL-4 and IFN-γ using enzyme-linked immunosorbent assay (ELISA), and to detect 25-(OH)-D using electrochemiluminescence method. The ELISA kit was provided by Shanghai Animalunion Biotechnology, and the cobas e 801 analytical unit for 25-(OH)-D testing was produced by Roche Company. All procedures were carried out according to the standard operation procedure.

### Statistical analysis

Using SPSS 26.0 software to conduct data analysis, specifically, count data was displayed by number of cases and percentages (%) and was compared using chi-square test. Measurement data was represented by $\overline{{\text{x}}} \pm {\text{s}}$ and was compared via one-way analysis of variance (ANOVA) between three groups. Using logistic regression analysis to conduct multivariate analysis of general data in MP asthma and MP non-asthma groups. Using ROC to analyze the predictive value of each variable regarding MP infection-related asthma. p<0.05 suggests that there is a significant difference.

## Results

### Comparison of the general data between three groups

The result suggested that there was not significant difference regarding gender, age, and BMI among three groups (*p* > 0.05). However, for recurrent upper respiratory tract infection and collective living, there were significant differences among three groups, and the number of children in the MP asthma group and MP non-asthma group was higher than that in the control group (*p* < 0.05). Table 1 presents the details.

**Table 1:** Comparison of general data between three groups.

General data	MP asthma group (*N* = 37)	MP non-asthma group (*N* = 41)	Control group (*N* = 34)	Statistics	*p*-value
Gender					
Male	20 (54.05%)	19 (46.34%)	18 (52.94%)	0.545	0.762
Female	17 (45.95%)	22 (53.66%)	16 (47.06%)		
Age	4.97 ± 0.95	4.78 ± 0.88	4.67 ± 0.73	1.807	0.341
BMI					
≤ 24	28 (75.68%)	34 (82.93%)	28 (82.35%)	0.771	0.680
> 24	9 (24.32%)	7 (17.07%)	6 (17.065%)		
Recurrent upper respiratory tract infection					
Yes	24 (64.86%)	17 (41.46%)	8 (23.53%)	12.439	0.02
No	13 (35.14%)	24 (58.54%)	26 (76.47%)		
Collective living					
Yes	30 (81.08%)	29 (70.73%)	14 (41.18%)	13.301	0.01
No	7 (18.92%)	12 (29.27%)	20 (58.82%)		

### Logistic analysis of general data in MP asthma and MP non-asthma groups

Regarding statistically significant variables comprising recurrent upper respiratory tract infection and collective living as independent variables, and the incidence of MP asthma and MP non-asthma as dependent variable to conduct multivariate logistic regression analysis. The result demonstrated that having history of recurrent upper respiratory tract infections (OR = 5.245, *p* < 0.05) and experiencing collective life (OR = 5.324, *p* < 0.05) were risk factors for MP infection-related asthma in children (Table 2). Similar results also presented in the MP non-asthma group, recurrent upper respiratory tract infections (OR = 2.079, *p* < 0.05) and collective life (OR = 3.261, *p* < 0.05) were identified as hazard factors of the appearance of MP without asthma (Table 3).

**Table 2:** Logistic analysis in MP Asthma.

Variables	β	SE	Wald	*p*-value	OR	95% CI
Recurrent upper respiratory tract infections	1.657	0.431	14.797	<0.001	5.245	2.254–12.202
Collective living	1.672	0.480	12.136	<0.001	5.324	2.078–13.640

**Table 3:** Multivariate analysis in MP non-asthma.

Variables	β	SE	Wald	*p*-value	OR	95% CI
Recurrent upper respiratory tract infections	0.732	0.335	4.759	0.029	2.079	1.077–4.011
Collective living	1.182	0.319	13.725	<0.001	3.261	1.745–6.095

### Comparison of 25-(OH)-D, IL-4, and IFN-γ between three groups

The significant differences were proved in the level of 25-(OH)-D, IL-4, and IFN-γ between three groups (*p* < 0.05). Compared with the control group, the level of 25-(OH)-D was significantly decreased in the case group, while IL-4 and IFN-γ showed opposite trends that were significantly increased in the case group. Additionally, the value of IFN-γ/IL-4 also significantly decreased in the case group, however, it was noticeable that there is no significant difference between MP asthma group and MP non-asthma group. Table 4 explains the details.

**Table 4:** Comparison of 25-(OH)-D, IL-4, and IFN-γ between three groups.

Variables	MP asthma group (*N* = 37)	MP non-asthma group (*N* = 41)	Control group (*N* = 34)	Statistics	*p*-value
25-(OH)-D (ng/ml)	22.59 ± 5.36^a, b^	25.73 ± 6.66^a^	30.96 ± 5.41	33.927	<0.001
IL-4 (pg/ml)	436.80 ± 133.44 ^a,b^	263.08 ± 119.61^a^	152.53 ± 44.65	122.918	<0.001
IFN-γ (pg/ml)	95.04 ± 20.97^a,b^	68.80 ± 24.76^a^	49.24.10 ± 25.34	49.978	<0.001
IFN-γ/IL-4	0.24 ± 0.11^a^	0.30 ± 0.15^a^	0.35 ± 0.20	5.715	0.004

^a^Compared with the control group, *p* < 0.05; ^b^Compared with the non-asthma group, *p* < 0.05

### The predictive value of single variable detection and joint detection for MP infection-related asthma

Using the occurrence of MP infection accompanied by asthma as the state variable (1 = asthma, 0 = non-asthma), and the value of 25-(OH)-D, IL-4, IFN-γ, and IFN-γ/IL-4 as test variables, corresponding ROC curves were plotted. The AUC was 0.765, 0.780, 0.853, 0.638, and 0.912 for 25-(OH)-D, IL-4, IFN-γ, IFN-γ/IL-4, and joint detection respectively. Among them, the AUC of joint detection was higher than others, and the sensitivity and specificity were 0.892 and 0.867 (Table 5).

**Table 5:** The value of variable in diagnosis of MP infection-related asthma.

Variables	Cut-off value	AUC	95% CI	Sensitivity (%)	Specificity (%)
25-(OH)-D	26.05ng/ml	0.765	0.687–0.843	0.811	0.674
IL-4	258.39 pg/ml	0.780	0.704–0.856	0.811	0.801
IFN-γ	74.95 pg/ml	0.853	0.797–0.909	0.865	0.707
IFN-γ/IL-4	0.19	0.638	0.548–0.728	0.541	0.793
Joint detection		0.912	0.868–0.957	0.892	0.867

## Discussions

MP is a common pathogen for community-acquired pneumonia (CAP) and other respiratory infection^33^. The general infection rate of MP ranges from 10–30%, while it can soar to 50% during epidemics^34^. Children, as the susceptible population of MP due to the underdeveloped immune system, show significant differences in the course, clinical manifestations, and complications when infected with MP^35,36^. The invasion of MP can cause severe damage not only affecting respiratory tract but also threatening other organs such as lung, heart, and liver^34^. It is evident that MP infection results in more significant and direct damage to the lung, numerous studies have demonstrated that MP infection may cause the symptom of asthma attack in children^37–39^, and can also induce acute asthma attack for children with pre-existing asthma^40–42^. Early diagnosis of MP infection is crucial to prevent disease progression and relevant complications. Nevertheless, the clinical diagnosis of MP infection, however, primarily relies on the detection of serological antibodies that displays an extremely high false negative rate^43^. Consequently, choosing a simple and efficient method to determine the diagnosis of MP infection has become a top priority.

Numerous studies have suggested that immune dysfunction and abnormal activation of humoral immunity are the basic pathogenesis of MP infection combined with asthma, and the imbalance between Th1 and Th2 cells is a noteworthy manifestation^11,44,45^. It is well known that there are evident differences regarding the function of Th1 and Th2 cells, concretely, Th1 cells mainly participate in cellular immunity via secreting IFN-γ and activating T cells and NK cells^46–48^, while Th2 mainly participate in humoral immunity via secreting IL-4 and stimulating B cells to product antibody^49,50^. Therefore, the value of IFN-γ/IL-4 represents whether the imbalance of Th1 and Th2 cells exists or not. The dominance of Th2 cells can be regarded as self-protection of body to alleviate the inflammatory response resulted from MP infection, to eliminate MP pathogens, and to minimize damage^51^. However, abundant antibodies produced under severe immune dysregulation condition can deposit in multiple organs such as the heart, lung, liver, brain, and kidney, leading to irreversible damage via activating complement system since there is common antigen between MP and human’s organs^52^. The study of Wang, Liang^53^, Li, Wang^54^, and Hu, Ye^55^ have suggested that MP infection can suppress the cellular immunity, while the humoral immunity is in an overactivated state, which denotes that the increase in IL-4 may be more significant compared to the increase in IFN-γ. Consistent with these previous studies, this study also demonstrated that MP infection can result in the obvious increase of IFN-γ and IL-4, specifically, the level in MP asthma group was significantly higher than that of non-asthma group and control group. Moreover, we also proved that the imbalance of immune function exists in children after MP infection, whilst there was no significant difference between MP asthma group and MP non-asthma group.

The role of 25-(OH)-D is diverse, such as regulating calcium and phosphorus metabolism, maintaining immune balance via affecting the differentiation and maturation of T cells, and affecting the occurrence and development of cancer^56,57^. Research has shown that the incidence of respiratory infectious diseases decreases with the increase of 25-(OH)-D, hence, supplementing 25-(OH)-D may serve as an effective method to prevent and treat respiratory diseases^58,59^. The finding of this study indicated that 25-(OH)-D in the case group whether with asthma or without asthma was lower compared with the control group, which can attribute to imbalanced diet and insufficient sunlight exposure. Moreover, the study of Zhou, Qiu^60^ pointed out that the lack of 25-(OH)-D can cause airway hyperresponsiveness by promoting the aggregation of eosinophils, leading to acute attacks of asthma or exacerbation of asthma symptoms. Consistent with previous results, current study also found a correlation between the level of 25-(OH)-D and the incidence of MP infection-related asthma, which further confirms that 25-(OH)-D can contribute to the emergence and development of MP infection-related asthma. Therefore, 25-(OH)-D should be considered as a necessary testing indicator for children with MP infection, and maintaining sufficient 25-(OH)-D can effectively reduce the incidence of asthma resulted from MP infection.

Additionally, this study also applied ROC curves to analyze the diagnostic value of 25-(OH)-D, IFN-γ, IL-4, IFN-γ/IL-4, and joint detection for MP infection. The results indicated that each variables show relatively high diagnostic ability focusing on MP-related asthma, and joint detection had the highest diagnostic ability. Therefore, in the early diagnosis of MP, it is necessary to consider the joint detection of multiple variables to maximize the sensitivity and specificity. Furthermore, the results of logistic regression analysis showed that recurrent upper respiratory tract infection and collective life are risk factors for MP infection in children. It is well known that the respiratory tract of patient with recurrent upper respiratory tract infections has weaker antibacterial ability and is more susceptible to multiple pathogen infection especially MP^61–63^. Moreover, collective life may increase the risk of cross infection with MP among children due to their undeveloped immune system^35^.

This study also has certain limitations. First, the number of cases is limited, reducing the reliability and accuracy of the results. Second, this study is an observational study, it is necessary to distinguish the course of MP infection and to dynamically compare changes in various indicators, which may contribute to develop more appropriate treatment measures.

## Conclusion

In summary, MP infection can lead to the significant increase in IFN-γ and IL-4, while the significant decrease in 25-(OH)-D in children, it is reasonable to assert that 25-(OH)-D plays an important protective effect in the process of MP infection. Regarding the diagnosis of MP asthma, the joint detection of 25-(OH)-D, IL-4, IFN-γ, and IFN-γ/IL-4 may improve diagnostic capabilities of MP infection-related asthma.

## Data Availability

The datasets used and/or analyzed during the current study available from the corresponding author on reasonable request.
